# 50. The great mimic fools again

**DOI:** 10.1093/rap/rky034.013

**Published:** 2018-09-20

**Authors:** Radhika Raghunath, Rahul Shah, Elaine Baguley

**Affiliations:** Rheumatology, Hull Royal Infirmary, Hull, UNITED KINGDOM

**Introduction:** We aim to present a rare case of osseous sarcoid in a 57 year old presenting with multiple sclerotic lesions of the axial skeleton with minimal involvement of other systems.

**Case description:** This 57 year old male initially presented in June 2016 to the neurosurgery department with acute onset of severe neck pain and brachialgia, following a twisting injury as part of his duties as a village care taker. A CT of the cervical spine demonstrated a C6/C7 disc prolapse and he was listed for a discectomy.

His past medical, family and drug history were unremarkable. He was a non-smoker who drank limited amounts of alcohol. In the past he had worked in an occupation that involved disposing of dead animals and exposure to animal faeces as part of his occupation as a refuse collector. Whilst awaiting this procedure, approximately a month later he developed severe subacute lower back pain which was non-inflammatory in nature but radiated down both legs. This prompted his general practitioner to organise an MRI of lumbo-sacral spine. Prior to the MRI being reported, this imaging was reviewed incidentally at a neurosurgical pre-operative clinic and it appeared to demonstrate multiple sclerotic lesions in thoracic and cervical spine suggestive of metastatic disease. Given the above findings, he was referred urgently to the cancer of unknown primary multi-disciplinary team for further assessment. Urinary Bence Jones protein and prostate-specific antigen were normal at this time. CRP was 2.4mg/L and the adjusted calcium was 2.34mmol/L, both also within normal limits. A CT thorax-abdomen and pelvis in January 2017 did not identify a primary malignancy but it did demonstrate indeterminate small pulmonary nodules and mediastinal lymphadenopathy. A bone scan did not show any uptake, and an attempted biopsy of the L4 vertebra was unsuccessful due to severe pain on contact with the periosteum. Further to this, a mediastinal node biopsy was organised via endoscopic ultrasound. In the interim, the gentleman had a PET-CT which showed high uptake in the symmetrical hilar and mediastinal adenopathy along with diffuse bone lesions involving the spine, acetabulum and sternum, raising the possibility of a sarcoid-like reaction secondary to malignancy. A sub-carinal biopsy subsequently demonstrated non-caseating epithelioid cell granulomas with no evidence of infective or malignant process, once again strongly suggestive of sarcoid. Granulomatous inflammation was also further demonstrated on a repeated attempt at bone marrow biopsy. He was then referred for a rheumatology opinion given the limited respiratory involvement in July 2017. Detailed history taking revealed a history of approximately 18 months of night sweats and insidious onset diffuse back pain. Due to his previous occupation an extensive infection screen was carried out including TB, toxoplasma, brucella, lyme and coxiella, all of which were negative. A serum ACE was 68 U/L (0-52 being the normal range). Following extensive serological testing for infective causes, he was started on prednisolone 40mg od in October 2017, which resulted in a dramatic improvement in his symptoms, namely back pain, fatigue and night sweats. Serum ACE normalised. The steroids were tapered after three months; however due to miscommunication the patient stopped it on Easter 2018. A few days later he developed florid synovitis affecting his left and right wrists along with his metacarpophalangeal joint. Steroids were recommenced and methotrexate started. A repeat PET-CT was organised, which confirmed significant interval regression in the intensity of FDG uptake in the spine with unchanged appearances of the lymphadenopathy. Further to this the gentleman rang the department to say he had stopped his methotrexate due to the development of a rash. He was also on amoxicillin for a presumed chest infection at the time. Due to the unclear association, DMARD therapy was changed to mycophenolate and he is currently on 500mg once a day, which is due to be titrated up to 1g.

**Discussion:** Up to 13% of patients with sarcoid present with radiologically evident skeletal involvement, but it is commonly appendicular. There are very few case report numbers of axial sarcoid as the primary presentation, especially sclerotic lesions. One of these reports a lady with Crohn’s and pelvic sarcoma, whilst the other had a preceding diagnosis of pulmonary sarcoid along with lytic and sclerotic bone lesions. Treatment initiation was also difficult due to the rarity of such a presentation and the extraneous factors such as his occupational history lending to significant diagnostic difficulty. The case is interesting not only due to its rarity but also the possibility of this being a paraneoplastic phenomena.

**Key Learning Points:** Multiple sclerotic lesions in a middle aged man are often a manifestation of metastatic malignancy. It is unusual to see axial sclerotic lesions as a primary manifestation of sarcoid, and it would be worthwhile lending credence to this differential in complex cases that don’t fit the typical pattern. It would be of great value for the authors to learn of any similar cases encountered by our peers and their management challenges.

**Disclosure: R. Raghunath:** None. **R. Shah:** None. **E. Baguley:** None.

